# Determination of the Antioxidant Capacity of Human Seminal Fluid Using a Fast and Accurate Electrochemical Approach

**DOI:** 10.3390/antiox15010035

**Published:** 2025-12-26

**Authors:** Youssef Hibaoui, Slim Khedhri, Dorothea Wunder, Josefina Vargas, Alexandre Vallée, Jean-Marc Ayoubi, Anis Feki

**Affiliations:** 1 Department of Obstetrics and Gynaecology, HFR Fribourg—Hôpital Cantonal, CH-1708 Fribourg, Switzerland; 2 Faculty of Science and Medicine, University of Fribourg, CH-1700 Fribourg, Switzerland; 3 Fertas Fribourg, HFR Fribourg—Hôpital Cantonal, CH-1708 Fribourg, Switzerland; 4 Department of Obstetrics and Gynaecology and Reproductive Medicine, Hôpital Foch-Faculté de Médecine Paris, 92150 Suresnes, France

**Keywords:** electrochemical approach, male infertility, oxidative stress, seminal plasma, total antioxidant capacity

## Abstract

Infertility affects around 10–15% of couples worldwide, out of which male factor contributes to 30–50% of cases of infertility. Oxidative stress, which corresponds to an imbalance between antioxidant capacities and reactive oxygen species, is considered a leading cause of male infertility. Therefore, the ability to monitor antioxidant capacity in seminal fluid is critical as it sustains free radical balance in the sperm. Most currently available methods to assess antioxidant capacity in seminal fluid are time-consuming, require specialized equipment, or are not easily implemented in clinical routine practice. Here, we evaluate the applicability of an electrochemical approach to determine the antioxidant capacity of human seminal fluid. We show that the results of this electrochemical approach are comparable to those of two reference methods for evaluating free radical scavenging activity, namely 2,20-azino-bis-3-ethylbenzothiazoline-6-sulfonic acid (ABTS) and 2,2-diphenyl-1-picrylhydrazyl (DPPH), when measuring the antioxidant capacity of seminal plasma or antioxidant molecules such as 6-hydroxy-2,5,7,8-tetramethylchromane-2-carboxylic acid (Trolox), ascorbic acid, and uric acid. Furthermore, we demonstrate the applicability of the method for the assessment of the antioxidant capacity of seminal fluid isolated from 30 normozoospermic patients (528.2 ± 142 nW). Further analysis demonstrates a positive correlation between the antioxidant capacity measured through the electrochemical approach and sperm concentration. Overall, this electrochemical approach provides a fast and accurate assessment of total antioxidant capacity in human seminal fluid. It may be implemented as a complementary tool in the routine evaluation of male infertility.

## 1. Introduction

Infertility is defined as a disease of the reproductive system characterized by failure to establish a clinical pregnancy after 12 months of regular, unprotected sexual intercourse or due to an impairment of a person’s capacity to reproduce, either as an individual or with his/her partner [1,2]. Male infertility accounts for approximately 30–50% of all cases of infertility. Among those, approximately half are considered idiopathic, while the rest of the infertility etiologies comprise congenital and acquired infertility [2,3,4]. Currently, the analysis of semen parameters according to the World Health Organization (WHO) represents the gold standard for male infertility diagnosis [5]. Recent efforts have been made to address the idiopathic causes of male infertility by clinical and research laboratories [4,6,7,8,9].

Several studies have suggested that oxidative stress, a condition characterized by an imbalance between reactive oxygen species (ROS) production and antioxidant defenses, plays a key role in male infertility [6,7,10,11]. Free radicals play an essential role in several physiological processes also called redox signaling processes [12]. In physiological amounts, ROS are beneficial for the maintenance of homeostasis in a variety of cellular functions [12], including sperm fertilization, chromatin compaction in maturing spermatozoa, motility, chemotaxis, sperm capacitation, hyperactivation, and acrosome reaction [7,13,14,15]. In this respect, ROS are maintained under physiological levels thanks to seminal antioxidants, which comprise both enzymatic antioxidants and non-enzymatic antioxidants [7,9,12]. The former, which includes superoxide dismutase, catalase, and glutathione peroxidase, removes ROS through catalytic activity, while the latter, such as α-tocopherol (vitamin E), ascorbic acid (vitamin A), uric acid, thiols, and others, scavenges or inactivates ROS. However, when ROS production overcomes the antioxidant capacities, sperm structure and functions can be affected, which in turn, leads to reproductive disorders, including fertilization failure, spontaneous miscarriage, and/or recurrent miscarriages [9,16,17]. At the cellular level, oxidative stress results in lipid peroxidation, protein changes, DNA damage, and sperm death, and this may lead to alterations in sperm quality, reducing sperm fertilizing potential [7,9,14,15].

Several methods have been developed to assess the total antioxidant capacity (TAC) of human biologic fluids, including blood, saliva and semen. Among these, 2,2-diphenyl-1-picrylhydrazyl (DPPH) and the 2,20-azino-bis-3-ethylbenzothiazoline-6-sulfonic acid (ABTS) free radical tests are the most commonly used [18,19,20,21]. The principle of these methods is based on the quantification of antioxidants present in a sample to prevent the oxidation of ABTS (or DPPH) to ABTS^•+^ (or DPPH^•^) radicals, resulting in a change in absorbance to a degree that is proportional to their concentrations. Then, the antioxidant capacity of the sample is compared with that of the standard antioxidant 6-hydroxy-2,5,7,8-tetramethylchromane-2-carboxylic acid or Trolox (a water-soluble tocopherol analog), and is therefore reported as a micromolar of Trolox equivalents. Even if a cutoff value of seminal plasma TAC levels has been proposed to distinguish between fertile and infertile men [22], TAC levels are not routinely assessed in standard infertility evaluation, mostly because this assay is cumbersome and costly with respect to equipment and skills [18,19,20,21,23]. Considering this, new approaches have aimed to characterize male infertility by monitoring seminal antioxidant capacity through simple, robust, and inexpensive techniques, which are needed in routine clinical settings. Recently, particular attention has been focused on the applicability of electrochemical methods to evaluate the antioxidant capacity of molecules and/or of biologic samples as an alternative to spectrophotometric methods. These electrochemical methods measure antioxidant capacity by quantifying electron transfers resulting from the reduction of oxidant compounds by antioxidants [24,25]. Among these electrochemical methods, voltammetry is generally more suitable for measuring redox processes and for evaluating antioxidant capacity [24,25,26]. Antioxidant capacity is determined by applying a controlled potential to a working electrode and measuring the resulting current, which is directly proportional to the amount of antioxidants present in the sample. Furthermore, electrochemical methods allow for the quick measurement of antioxidant capacity without modifying the sample’s native conditions and offer high sensitivity [24,25,26].

In this study, we tested the applicability of an electrochemical-based technique to determine the antioxidant capacity of human seminal fluid. First, we investigated the antioxidant capacity of the most common antioxidants present in biological fluids including Trolox, ascorbic acid, and uric acid using the electrochemical approach and two reference methods for evaluating free radical scavenging activity, namely the ABTS and DPPH assays. Then, the antioxidant capacity of seminal fluid from 30 normozoospermic individuals was measured and analyzed using the electrochemical approach. As for the antioxidant molecules, linear sweep voltammograms (LSVs) and pseudo-titrated voltammograms (PTVs) were generated and analyzed for these seminal plasma samples. Finally, we examined the correlations between the electrochemical antioxidant capacity of seminal plasma samples and semen parameters, including sperm concentration, total motility, progressive motility, and normal sperm morphology.

## 2. Materials and Methods

### 2.1. Study Population

From March 2022 until August 2024, 30 males from couples seeking fertility treatment at the Department of Obstetrics and Gynaecology of the University Hospital of Fribourg—HFR Fribourg were invited to participate in the study. Eligible participants were between 29 and 63 years old, with a planned semen analysis in the context of a standard infertility diagnostic workup. This study was performed according to the principles of the Declaration of Helsinki and approved by the Institutional Ethics Board of CER-VD (Commission Cantonale d’Ethique de la recherche sur l’être humain; 2017-01170). All participants provided written informed consent for their participation in the study.

### 2.2. Assessment of Sperm Quality and Seminal Plasma Sample Preparation

Semen samples were collected in the laboratory via masturbation after a median period of 3 days of sexual abstinence (see Table 1 for the clinical characteristics of study population). Samples were incubated 20–40 min at 37 °C to allow for liquefaction and then thoroughly homogenized using a disposable Pasteur pipette. Sperm concentration and motility were measured using the computer-assisted sperm analyzer QualiSperm (AKYmed, Morges, Switzerland). When the concentration was up to 40.10^6^/mL, commercialized HEPES-buffered solution (SpermWash, Gynotec, Malden, The Netherlands) was used to dilute the native sperm. Aliquots of 10 µL of the diluted or non-diluted sample were transferred into a 20 µm deep counting chamber (20 µm—2 chambers slide, Leja Products, GN Nieuw Vennep, The Netherlands) that was placed on a microscope equipped with a heated stage. Sperm concentration and kinetic parameters were recorded and the video sequences stored. The semen parameters were analyzed according to the WHO standards [5], and are reported in Table 1. For further analysis, 1 mL of semen sample was centrifuged for 5 min at 500× *g* and the plasma was removed, either to be analyzed fresh or after freezing and storage at −80 °C. The first measurement of antioxidant capacity using the electrochemical approach based on pH was performed on fresh seminal plasma.

### 2.3. Electrochemical Antioxidant Capacity Measurements

The antioxidant capacity of seminal plasma was measured by a commercial EDEL electrochemical biosensor, the EDEL meter (Edel therapeutics, Lausanne, Switzerland), as previously described [27,28]. This device includes a biological detector coupled with a chemical transducer and a specific software that can be run by a computer. The working electrode was a screen-printed carbon electrode operated in conjunction with a screen-printed counter/reference electrode (Ag/AgCl, CE/RE). All potentials were measured with respect to Ag/AgCl CE/RE. For each electrochemical measurement, 2 µL of a sample (seminal plasma or antioxidant molecule) was deposited onto a fresh electrode strip. Linear sweep voltammograms (LSVs) were recorded within less than 10 secondes after sample loading from 0 to 1.2 V with a scan rate of 100 mV/s under ambient conditions. Pseudo-titrated voltammograms (PTVs) were also generated by the software. The total antioxidant capacity (also called antioxidant power), expressed in nanowatts, was calculated as the integral of the PTVs [27,28]. The value of the area under the curve of the PTVs was shown to be a better indicator of antioxidant capacity than the peak of the PTVs [27,29,30]. A fresh working electrode was used for each sample measurement.

### 2.4. Antioxidant Capacity Determination by DPPH Assay

The 2,2-diphenyl-1-picrylhydrazyl (DPPH) free radical scavenging activity was determined by a method previously described [31], and adapted to seminal plasma samples. Briefly, 10 µL of diluted seminal plasma samples (or antioxidant molecules) was mixed with 190 µL of DPPH solution (100 µM in methanol) in each well of a 96-well plate. Then, the mixture was incubated for 30 min in the dark at room temperature, after which the absorbance of the solution was measured at 517 nm (corresponding to the maximum absorption), using Varioskan LUX Multimode Microplate Reader (Thermofisher, Allschwil, Switzerland). Antioxidants present in the seminal plasma sample reduced the concentration of DPPH^•^ radicals, leading to solution decolorization from violet to yellow, to a degree proportional to their concentration. The percentage of radical scavenging activity (expressed as % RSA) was calculated as follows: RSA (%) = [(Control absorbance − Sample absorbance)/Control absorbance] × 100. In parallel, standard curves were established using concentrations between 0.0 and 1.0 mmol/L of 6-hydroxy-2,5,7,8-tetramethylchromane-2-carboxylic acid (Trolox), ascorbic acid, and uric acid.

### 2.5. Antioxidant Capacity Determination by ABTS Assay

The ABTS assay is based on the ability of the antioxidants present in a sample to scavenge the 2,20-azino-bis(3-ethylbenzothiazoline-6-sulfonic acid) (ABTS) radical (ABTS^•+^), which is enzymatically pre-generated by reacting a 7 mM ABTS stock solution with 2.45 mM potassium persulfate and allowing the mixture to stand in the dark overnight at room temperature [32]. After performing this assay, the ABTS^•+^ solution was diluted in phosphate-buffered saline (PBS), which had a pH of 7.4, to reach an absorbance of ~0.70 at 734 nm. For the microplate ABTS assay, 10 µL of diluted seminal plasma sample (or antioxidant molecules) was mixed with 190 µL of the diluted ABTS^•+^ solution in each well of a 96-well plate. The ABTS^•+^ radical has a blue-green color, with a maximum absorption at 734 nm; therefore, the change in absorbance was measured at this wavelength using Varioskan LUX Multimode Microplate Reader (Thermofisher, Allschwil, Switzerland). Antioxidants in the sample reduced the concentration of the ABTS^•+^ radical, leading to solution decolorization to a degree proportional to their concentration. The percentage of radical scavenging activity (expressed as % RSA) was calculated as follows: RSA (%) = [(Control absorbance − Sample absorbance)/Control absorbance] × 100. In parallel, standard curves were performed using concentrations between 0.0 and 1.0 mmol/L of 6-hydroxy-2,5,7,8-tetramethylchromane-2-carboxylic acid (Trolox), ascorbic acid, and uric acid.

### 2.6. Statistical Analysis

Graphs were constructed, and the data were analyzed using GraphPad Prism 9 software (GraphPad, San Diego, CA, USA). The normality of distribution for all parameters presented in Table 1 was analyzed with the Shapiro–Wilk test and reported as median values and interquartile ranges (IQRs). Linear relationships between antioxidant capacity (nW) and antioxidant concentrations, as well as between radical scavenging activity (%RSA) and antioxidant concentrations, were assessed using simple linear regression. The regression equation and R^2^ were reported. Correlations between continuous variables were assessed using Spearman’s rank correlation coefficient. Two-sided *p* values ≤ 0.05 were considered statistically significant.

## 3. Results

### 3.1. Assessment of the Antioxidant Capacity of Trolox, Uric Acid and Ascorbic Acid Using the Electrochemical Approach

**Figure 1:**
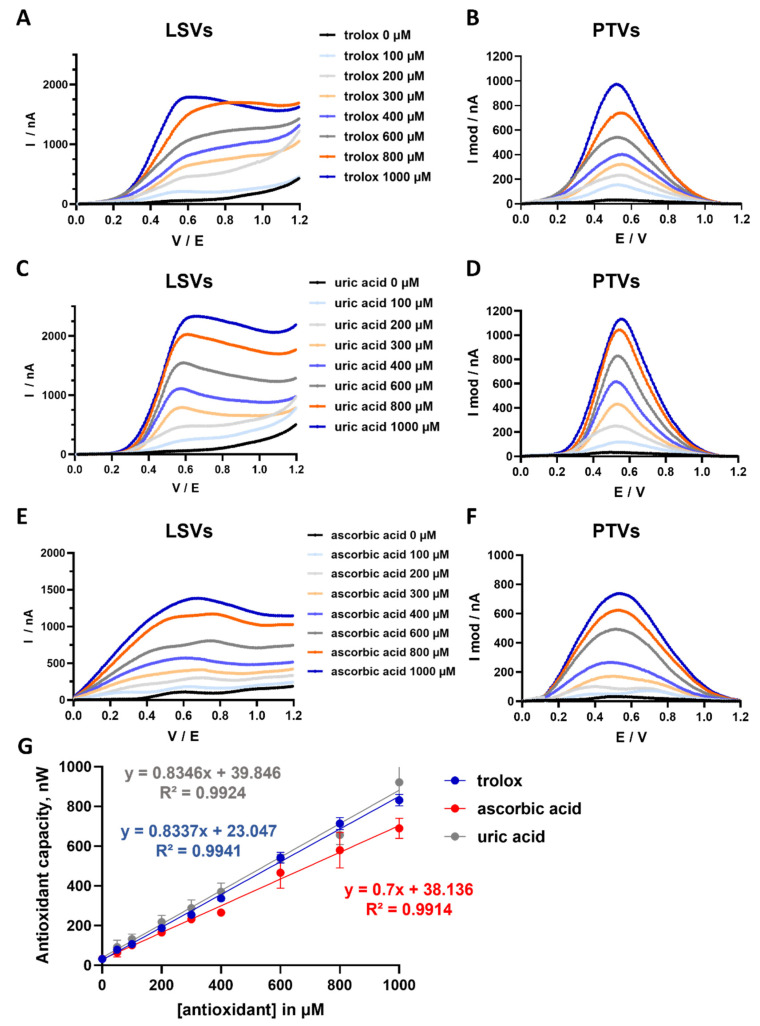
Assessment of the antioxidant capacity of Trolox, ascorbic acid and uric acid using the electrochemical approach. Typical linear sweep voltammograms (LSVs) are shown for concentrations of Trolox (in (**A**)), uric acid (in (**C**)) and ascorbic acid (in (**E**)), ranging from 0 to 1000 µM. These LSVs were recorded from 0 V to 1.2 V, with a scan rate of 100 mV/s under ambient conditions. Typical pseudo-titrated voltammograms (PTVs) are shown for concentrations of Trolox (in (**B**)), uric acid (in (**D**)) and ascorbic acid (in (**F**)), ranging from 0 to 1000 µM. The antioxidant capacities of Trolox, ascorbic acid and uric acid have been calculated as the integrals of the PTVs (see material and methods) and are expressed in nanowatts (nW), showing a linear relationship between the antioxidant capacity of these antioxidant molecules and their concentrations (in (**G**)). Data are presented as means ± SDs from 6 independent experiments.

We first investigated the antioxidant capacity of several antioxidant molecules present in the biological fluids, including 6-hydroxy-2,5,7,8-tetramethylchromane-2-carboxylic acid (Trolox, a water-soluble tocopherol analog), uric acid, and ascorbic acid, using an electrochemical approach. As shown in Figure 1, linear sweep voltammograms (LSVs) and pseudo-titrated voltammograms (PTVs) were generated and analyzed for several concentrations of Trolox (Figure 1A,B), ascorbic acid (Figure 1C,D), and uric acid (Figure 1E,F). In this respect, the value of the area under the curve of the PTVs showed itself to be a better indicator of total antioxidant capacity than the peak of the PTVs alone because the former accounts for the cumulative effect of all antioxidant molecules in a sample, providing a more comprehensive assessment of antioxidant activity [29,30]. Importantly, the values of the area under the curve of the PTVs (expressed in nanowatts) were proportional to the concentrations of Trolox (Figure 1B,G). Similar linear relationships were found between the values of the area under the curve of the PTVs, indicative of total antioxidant capacity with uric acid (Figure 1D,G) or ascorbic acid concentrations (Figure 1F,G). Altogether, these results support the applicability of the technique in determining the antioxidant capacity of a solution, as well as the possibility to evaluate the concentration of antioxidant molecules based on the assessment of the titration of the antioxidant capacity using this electrochemical approach.

### 3.2. Assessment of the Antioxidant Capacity of Trolox, Uric Acid and Ascorbic Acid Using the ABTS and DPPH Assays

**Figure 2:**
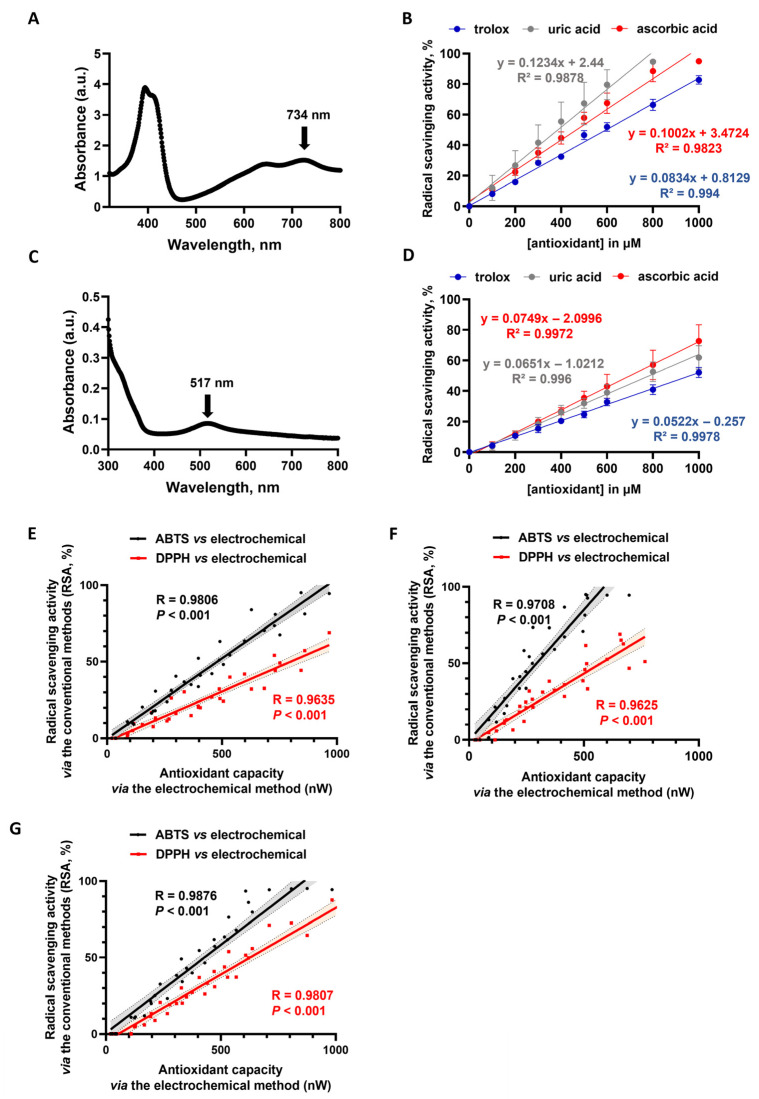
Assessment of the antioxidant capacity of Trolox, ascorbic acid and uric acid using the DPPH and ABTS assay. (**A**) Absorbance spectra of 2,20-Azino-bis-3-ethylbenzothiazoline-6-sulfonic acid (ABTS), showing maximum absorbance at 734 nm. (**B**) Radical scavenging activity of the antioxidants Trolox, ascorbic acid, and uric acid for concentrations ranging from 0 to 1000 µM determined using the ABTS assay at a wavelength of 734 nm. (**C**) Absorbance spectra of and 2,2-diphenyl-1-picrylhydrazyl (DPPH) showing maximum absorbance at 517 nm. (**D**) Radical scavenging activity of the antioxidants Trolox, ascorbic acid, and uric acid for concentrations ranging from 0 to 1000 µM determined using the DPPH assay at a wavelength of 517 nm. Spearman correlations between the antioxidant capacity assessed by the electrochemical approach and the radical scavenging activity (% RSA) assessed with the conventional ABTS and DPPH assays for Trolox (in (**E**)), ascorbic acid (in (**F**)), and uric acid (in (**G**)). The Spearman r value and the *p* value are also shown. Color-shaded areas indicate 95% confidence intervals. Data are presented as means ± SDs from 4 independent experiments.

The antioxidant capacities of the same antioxidant molecules were then measured using two reference methods for evaluating free radical scavenging activity, namely 2,20-azino-bis-3-ethylbenzothiazoline-6-sulfonic acid (ABTS) and 2,2-diphenyl-1-picrylhydrazyl (DPPH) (Figure 2). The absorption maximum for ABTS is presented in Figure 2A. The ABTS assay is based on the decrease in the absorbance of ABTS^•+^ at a wavelength of 734 nm when it reacts with antioxidants present in the sample. As expected, this method allowed for the determination of the antioxidant capacity of ascorbic acid, Trolox, and uric acid (Figure 2B) [18,19,20,21]. As shown in Figure 2B, a linear relationship was observed between the percentage of radical scavenging activity (expressed as % RSA) and the antioxidant molecule concentration (0–1000 µM) using the ABTS assay.

Moreover, the antioxidant capacity of the same antioxidant molecules was evaluated using the DPPH assay. This assay is based on the decrease in the absorbance of the DPPH^•^ radical at a wavelength of 517 nm (Figure 2C) when it reacts with antioxidants present in the sample. Consistent with previous studies, this method allowed for the determination of the antioxidant capacity of ascorbic acid, Trolox, and uric acid (Figure 2B) [18,19,20,21]. As shown in Figure 2D, a linear relationship was observed between the percentage of radical scavenging activity (expressed as % RSA) and the antioxidant molecule concentration (0–1000 µM) using the DPPH assay.

Finally, correlations between the conventional methods (ABTS and DPPH) and the electrochemical approach were investigated using the antioxidant molecules Trolox, ascorbic acid and uric acid (Figure 2E–G). Importantly, the antioxidant capacity assessed by using the electrochemical approach exhibited strong positive correlations with the radical scavenging activity (% RSA) assessed by the conventional ABTS and DPPH assays, regardless of whether the antioxidant molecule investigated was Trolox, ascorbic acid or uric acid (Figure 2E–G). Altogether, these results demonstrate the comparable applicability of the electrochemical approach in evaluating the antioxidant capacity of antioxidant molecules to that of the conventional ABTS and DPPH assays.

### 3.3. Antioxidant Capacity of Seminal Plasma from Normozoospermic Individuals Determined Using the Electrochemical Approach

**Figure 3:**
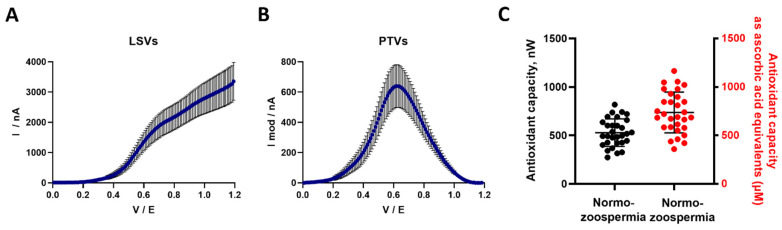
Assessment of the antioxidant capacity of human semen using the electrochemical approach. (**A**) Representative linear sweep voltammogram (LSV) traces are shown for seminal plasma from normozoospermic patients. These LSVs were recorded from 0 to 1.2 V with a scan rate of 100 mV/s under ambient conditions. (**B**) Representative pseudo-titrated voltammogram (PTV) traces are shown for seminal plasma from normozoospermic patients. (**C**) Antioxidant capacity is expressed in nanowatts (in nW, left *Y*-axis) and as vitamin C equivalents (in µM, right *Y*-axis) of semen from 30 normozoospermic patients. Data are presented as means ± SDs for 30 normozoospermic patients.

Antioxidant capacity was then measured in seminal fluid from normozoospermic patients using the electrochemical approach. The semen parameters were analyzed according to the WHO standards [5], and are reported in Table 1. The median age and period of abstinence were 39.01 years (IQR 32.61–40.67) and 3 days (IQR 2–4.25), respectively (Table 1). As shown in Figure 3A,B, LSVs and PTVs were generated and analyzed, showing a mean value of 528.2 ± 142 nW for antioxidant capacity in seminal plasma from normozoospermic patients (Figure 3C). Thus, these results revealed large interindividual differences in terms of semen antioxidant capacity values (from 273.0 to 817 nW) (Figure 3C), consistent with the unequal level of spermatozoa protection against ROS and oxidative stress among the normozoospermic patients. Considering that ascorbic acid is the main antioxidant molecule in human seminal fluid [33,34], and that most of the seminal antioxidant capacity values assessed by the conventional methods are expressed as vitamin C equivalents (or as Trolox equivalents), we also expressed the antioxidant capacity measured by the electrochemical approach as vitamin C equivalents. Thus, the antioxidant capacity of seminal plasma from normozoospermic patients was 0.736 ± 0.211 mM ascorbic acid equivalents.

**Figure 4:**
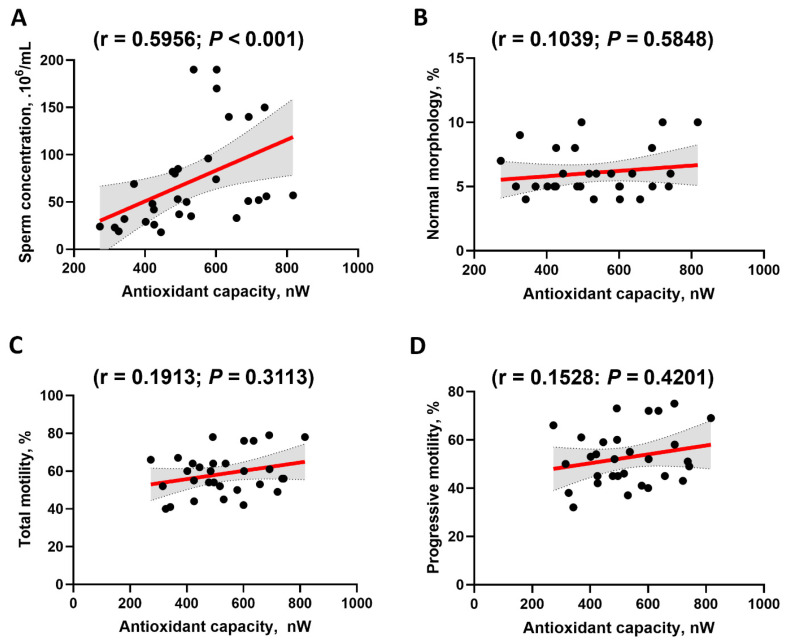
Correlations between electrochemical antioxidant capacity values and semen parameters. (**A**) Spearman correlations between antioxidant capacity, assessed by the electrochemical approach, and the sperm concentration (expressed as .10^6^/mL). (**B**) Spearman correlations between antioxidant capacity, assessed by the electrochemical approach, and normal morphology (expressed as a percentage). (**C**) Spearman correlations between antioxidant capacity, assessed by the electrochemical approach, and total motility (expressed as a percentage). (**D**) Spearman correlations between antioxidant capacity, assessed by the electrochemical approach, and progressive motility (expressed as a percentage). The Spearman r value and the *p* value are also shown. Color-shaded areas indicate 95% confidence intervals.

Further analysis revealed a significant positive Spearman correlation between the antioxidant capacity measured through the electrochemical approach and the sperm concentration (r = 0.5956; *p* < 0.001) (Figure 4). No significant correlations were observed between the antioxidant capacity and total motility, progressive motility or normal morphology (all *p* > 0.30), although the confidence intervals were wide due to the limited sample size and the restricted range of semen parameters in normozoospermic populations (Figure 4). Collectively, these results demonstrate the applicability of the electrochemical approach in evaluating the antioxidant capacity of human seminal plasma.

## 4. Discussion

Approximately 30–50% of all cases of infertility occur in males, and half of these cases are considered idiopathic [2,3,4]. Because the etiology of idiopathic male infertility cannot be determined, current treatments are based mainly on assisted reproductive technology or empirical medical therapies, including lifestyle modifications, as well as hormonal and non-hormonal therapies [2,4]. However, among the reported etiologies, the involvement of oxidative stress as a cause of male infertility is supported by elevated seminal oxidation reduction potential in 80% of men with infertility [6,7,8,10,11]. Since oxidative stress arises because of an imbalance between ROS production and antioxidant capacity, the evaluation of the total antioxidant capacity of human seminal fluid is of clinical interest in the workup of male infertility [2,6,13,15].

Several assays have been developed to evaluate the antioxidant capacity of human seminal fluid that consider the involvement of oxidative stress in male infertility [18,19,20,21]. As 2,20-azino-bis-3-ethylbenzothiazoline-6-sulfonic acid (ABTS) and 2,2-diphenyl-1-picrylhydrazyl (DPPH) assays are affordable, these are the most extensively exploited methodologies for measuring free radical scavenging activity. However, these assays are cumbersome and require expensive equipment and technical skills. In addition, both assays have been criticized for their lack of biological relevance, because both radicals do not exist in nature, and for the absence of standardization across different steps of the protocol in them [20,21,35]. Different antioxidant capacity values may be obtained for some antioxidant molecules, depending on the way in which ABTS^•+^ is generated (metmyoglobin/hydrogen peroxide or potassium persulfate). Other factors like the reaction time and the intrinsic antioxidant activity of samples have been shown to affect the results: some antioxidants react completely and very quickly with radicals, while others react slowly (or show a combination of fast and slow reactions). Thus, water, solvent, pH, and light exposure may affect the evaluation of antioxidant capacity based on the change in DPPH^•^ absorbance [20,23]. Recently, particular interest has been shown in the use of electrochemical-based methods to evaluate the antioxidant capacity of molecules or of biologic samples given their advantages, which include practicality, speed, and sensitivity [24,25,26,27,28]. Interestingly, the antioxidant capacity assessed by the electrochemical approach showed strong positive correlations with the radical scavenging activity assessed by the conventional ABTS and DPPH assays, regardless of the antioxidant molecule investigated. In addition, compared to the conventional analytical methods, i.e., DPPH and ABTS assays, the proposed electrochemical approach displayed several advantages that could be of interest in routine clinical practice. Our assay can be performed directly after semen sample collection within a few minutes with simple, portable, and inexpensive instrumentation; after a few minutes of centrifugation to obtain semen plasma, a duration of less than 30 secondes is required to assess semen antioxidant capacity. Thus, our method requires only a small volume of sample as a semen volume of two microliters was enough to assess semen antioxidant capacity.

Several other observations that emerged from the current study support the potential clinical use of this electrochemical approach. Consistent with a previous report, this method allowed the determination of the antioxidant capacity of most common antioxidants present in biological fluids including ascorbic acid, uric acid, and a derivative of vitamin E [27,28]. Here, the integral of the pseudo-titrated voltammogram (PTV) was used to calculate the antioxidant capacity as the value of the area under the curve of the PTVs was shown to be a better indicator of the total antioxidant capacity of a sample than the peak of PTVs [29,30]. As expected, the values of the area under the curve of the PTVs (indicative of the total antioxidant capacity) were proportional to the concentrations of the antioxidant molecules investigated (Figure 1). In previous studies, this electrochemical method was used to determine the antioxidant capacity of human saliva and blood [27,28], but our study is the first to assess the antioxidant capacity of human seminal fluid samples using this method. In this regard, the higher antioxidant capacity of human seminal fluid (~528 nW) measured in our study compared with that of saliva and blood samples (~280 nW and ~79 nW, respectively) [27,28] agrees with the results of a recent study showing a higher antioxidant profile for human seminal fluid compared to saliva and blood using the ABTS method [36]. In fact, spermatozoa are especially vulnerable to oxidative stress due to their limited ability to counteract excessive ROS. The reduced cytoplasmic volume in spermatozoa (which limits the storage of antioxidant defense mechanisms) and the high concentration of polyunsaturated fatty acids in their plasma membrane compared to that in oocytes or somatic cells make them particularly sensitive to ROS-induced damage [7,9]. Consequently, spermatozoa are only dependent on the availability of antioxidants in the extracellular compartment. Another important observation is the large interindividual differences in semen antioxidant capacity values (from 273.0 to 817 nW; median 506.5 nW), the mean of which was 528.2 ± 142 nW for the normozoospermic patients. These results suggest an unequal level of spermatozoa protection against ROS and oxidative stress among these normozoospermic patients. Moreover, when the semen antioxidant capacity assessed through the electrochemical approach was expressed as vitamin C equivalents, a mean antioxidant capacity value of 0.736 ± 0.211 mM ascorbic acid equivalents was found. These results contrast with previously published values, as a mean value of 1.4 mM ascorbic acid equivalents was found in normozoospermic patients using the conventional TAC/ABTS method [36,37]. However, these discrepancies may be attributable to the methodologies used for the assessment of seminal antioxidant capacity or to the greater proportion of seminal plasma samples with lower antioxidant capacity among our patient cohort. Finally, further analysis revealed a positive correlation between the antioxidant capacity measured through the electrochemical approach and the sperm concentration, but no correlations were found with the other semen parameters. These results are consistent with previous studies showing reduced sperm quality with reduced antioxidant capacity [22,37], and support the idea that electrochemical antioxidant capacity can be used as a marker in sperm quality evaluation. Collectively, these observations outline the key role of antioxidants in protecting sperm cells from oxidative stress and the importance of their evaluation in routine clinical settings involving spermatozoal dysfunction.

Despite the relevant results, some limitations are present in the current study. First, the sample size was relatively small (n = 30) and comprised only normozoospermic participants. Thus, the broad age range among participants (median: 39.04; IQR: 32.61–40,67 in years) may represent an additional limitation, since age influences semen parameters [38,39]. Given the limited sample size and the exploratory nature of the analyses, no formal adjustment for multiple testing was applied. Therefore, these findings should be interpreted with caution and require confirmation in larger, independent cohorts. Thus, larger studies including men with impaired sperm quality are required to confirm the present observations. Accordingly, the absence of significant correlations between the antioxidant capacity of the human seminal samples measured with this electrochemical-based approach and the seminal quality parameters (normal morphology, total and progressive motility) should be interpreted with caution as this may reflect sampling limitations rather than the absence of biological effects. Further studies integrating a larger cohort of normozoospermic individuals and patients with low sperm concentrations (oligozoospermia), low percentages of normal sperm morphology (teratozoospermia) and low percentages of total and progressive motility (asthenozoospermia) will confirm and extend the present findings.

## 5. Conclusions

In conclusion, our study supports the applicability of the electrochemical-based approach in the assessment of the antioxidant capacity of human seminal fluid. The critical need to develop diagnostic tools for the assessment of oxidative stress in male infertility and the numerous advantages of our electrochemical-based approach, including its practicality and speed, make it a valid alternative for the determination of the antioxidant capacity of human semen. Such assessments may be of particular interest in the field of male infertility, where an imbalance between ROS production and the antioxidant capacity of spermatozoa has been linked to human sperm dysfunction [6,7,8,10,11]. However, further studies are needed to apply this tool in the diagnosis and medical care of infertile patients. Of note is that ongoing investigations aimed at comparing the antioxidant capacity of seminal fluid from normozoospermic individuals with that of seminal fluids from patients with oligozoospermia, teratozoospermia, and asthenozoospermia using this method will indicate whether this electrochemical-based approach fulfills its potential.

## Figures and Tables

**Table 1:** Clinical characteristics of study population.

Parameter (Normospermia, n = 30)	Median	Interquartile Range (IQR)
Age, years	39.04	(32.61–40.67)
Period of abstinence, days	3	(2–4.25)
pH	7.5	(7.5–7.7)
Sperm concentration .10^6^/mL	52.5	(32.75–87.75)
Total motility, %	58.0	(51.5–64.5)
Progressive motility, %	51.5	(44.50–60.25)
Normal morphology, %	5	(5.00–6.25)

The values are presented as medians and interquartile ranges (IQRs).

## Data Availability

All data are contained within the article. Further inquiries can be directed to the corresponding authors.

## References

[1] Zegers-Hochschild F., Adamson G.D., Dyer S., Racowsky C., de Mouzon J., Sokol R., Rienzi L., Sunde A., Schmidt L., Cooke I.D. (2017). The International Glossary on Infertility and Fertility Care, 2017. Fertil. Steril..

[2] Eisenberg M.L., Esteves S.C., Lamb D.J., Hotaling J.M., Giwercman A., Hwang K., Cheng Y.-S. (2023). Male infertility. Nat. Rev. Dis. Primers.

[3] Chehab M., Madala A., Trussell J.C. (2015). On-label and off-label drugs used in the treatment of male infertility. Fertil. Steril..

[4] Agarwal A., Baskaran S., Parekh N., Cho C.-L., Henkel R., Vij S., Arafa M., Panner Selvam M.K., Shah R. (2021). Male infertility. Lancet.

[5] WHO (2021). WHO Laboratory Manual for the Examination and Processing of Human Semen.

[6] Agarwal A., Parekh N., Panner Selvam M.K., Henkel R., Shah R., Homa S.T., Ramasamy R., Ko E., Tremellen K., Esteves S. (2019). Male Oxidative Stress Infertility (MOSI): Proposed Terminology and Clinical Practice Guidelines for Management of Idiopathic Male Infertility. World J. Mens Health.

[7] Evans E.P.P., Scholten J.T.M., Mzyk A., Reyes-San-Martin C., Llumbet A.E., Hamoh T., Arts E.G.J.M., Schirhagl R., Cantineau A.E.P. (2021). Male subfertility and oxidative stress. Redox Biol..

[8] Aitken R.J., Drevet J.R., Moazamian A., Gharagozloo P. (2022). Male Infertility and Oxidative Stress: A Focus on the Underlying Mechanisms. Antioxidants.

[9] Bouhadana D., Godin Pagé M.-H., Montjean D., Bélanger M.-C., Benkhalifa M., Miron P., Petrella F. (2025). The Role of Antioxidants in Male Fertility: A Comprehensive Review of Mechanisms and Clinical Applications. Antioxidants.

[10] Agarwal A., Rana M., Qiu E., AlBunni H., Bui A.D., Henkel R. (2018). Role of oxidative stress, infection and inflammation in male infertility. Andrologia.

[11] Bisht S., Faiq M., Tolahunase M., Dada R. (2017). Oxidative stress and male infertility. Nat. Rev. Urol..

[12] Sies H., Berndt C., Jones D.P. (2017). Oxidative Stress. Annu. Rev. Biochem..

[13] Du Plessis S.S., Agarwal A., Halabi J., Tvrda E. (2015). Contemporary evidence on the physiological role of reactive oxygen species in human sperm function. J. Assist. Reprod. Genet..

[14] Nowicka-Bauer K., Nixon B. (2020). Molecular Changes Induced by Oxidative Stress that Impair Human Sperm Motility. Antioxidants.

[15] Castleton P.E., Deluao J.C., Sharkey D.J., McPherson N.O. (2022). Measuring Reactive Oxygen Species in Semen for Male Preconception Care: A Scientist Perspective. Antioxidants.

[16] Kamkar N., Ramezanali F., Sabbaghian M. (2018). The relationship between sperm DNA fragmentation, free radicals and antioxidant capacity with idiopathic repeated pregnancy loss. Reprod. Biol..

[17] Opuwari C.S., Henkel R.R. (2016). An Update on Oxidative Damage to Spermatozoa and Oocytes. BioMed Res. Int..

[18] Gupta S., Finelli R., Agarwal A., Henkel R. (2021). Total antioxidant capacity—Relevance, methods and clinical implications. Andrologia.

[19] Ilyasov I.R., Beloborodov V.L., Selivanova I.A., Terekhov R.P. (2020). ABTS/PP Decolorization Assay of Antioxidant Capacity Reaction Pathways. Int. J. Mol. Sci..

[20] Munteanu I.G., Apetrei C. (2021). Analytical Methods Used in Determining Antioxidant Activity: A Review. Int. J. Mol. Sci..

[21] Christodoulou M.C., Orellana Palacios J.C., Hesami G., Jafarzadeh S., Lorenzo J.M., Domínguez R., Moreno A., Hadidi M. (2022). Spectrophotometric Methods for Measurement of Antioxidant Activity in Food and Pharmaceuticals. Antioxidants.

[22] Mahfouz R., Sharma R., Sharma D., Sabanegh E., Agarwal A. (2009). Diagnostic value of the total antioxidant capacity (TAC) in human seminal plasma. Fertil. Steril..

[23] Schaich K.M., Tian X., Xie J. (2015). Hurdles and pitfalls in measuring antioxidant efficacy: A critical evaluation of ABTS, DPPH, and ORAC assays. J. Funct. Foods.

[24] Haque M.A., Morozova K., Ferrentino G., Scampicchio M. (2021). Electrochemical Methods to Evaluate the Antioxidant Activity and Capacity of Foods: A Review. Electroanalysis.

[25] Sochor J., Dobes J., Krystofova O., Ruttkay-Nedecky B., Babula P., Pohanka M., Jurikova T., Zitka O., Adam V., Klejdus B. (2013). Electrochemistry as a Tool for Studying Antioxidant Properties. Int. J. Electrochem. Sci..

[26] Pisoschi A.M., Cimpeanu C., Predoi G. (2015). Electrochemical Methods for Total Antioxidant Capacity and its Main Contributors Determination: A review. Open Chem..

[27] Tacchini P., Lesch A., Neequaye A., Lagger G., Liu J., Cortés-Salazar F., Girault H.H. (2013). Electrochemical Pseudo-Titration of Water-Soluble Antioxidants. Electroanalysis.

[28] Bardyn M., Maye S., Lesch A., Delobel J., Tissot J.-D., Cortés-Salazar F., Tacchini P., Lion N., Girault H.H., Prudent M. (2017). The antioxidant capacity of erythrocyte concentrates is increased during the first week of storage and correlated with the uric acid level. Vox Sang..

[29] Schilder W.H., Tanumihardja E., Leferink A.M., van den Berg A., Olthuis W. (2020). Determining the antioxidant properties of various beverages using staircase voltammetry. Heliyon.

[30] Chevion S., Roberts M.A., Chevion M. (2000). The use of cyclic voltammetry for the evaluation of antioxidant capacity. Free Radic. Biol. Med..

[31] Blois M.S. (1958). Antioxidant Determinations by the Use of a Stable Free Radical. Nature.

[32] Re R., Pellegrini N., Proteggente A., Pannala A., Yang M., Rice-Evans C. (1999). Antioxidant activity applying an improved ABTS radical cation decolorization assay. Free Radic. Biol. Med..

[33] Lazzarino G., Listorti I., Muzii L., Amorini A.M., Longo S., Di Stasio E., Caruso G., D’Urso S., Puglia I., Pisani G. (2018). Low-molecular weight compounds in human seminal plasma as potential biomarkers of male infertility. Hum. Reprod..

[34] Lewis S.E.M., Sterling E.S.L., Young I.S., Thompson W. (1997). Comparison of individual antioxidants of sperm and seminal plasma in fertile and infertile men. Fertil. Steril..

[35] Prior R.L., Wu X., Schaich K. (2005). Standardized Methods for the Determination of Antioxidant Capacity and Phenolics in Foods and Dietary Supplements. J. Agric. Food Chem..

[36] Aitken R.J., Wilkins A., Harrison N., Bahrami M., Gibb Z., McIntosh K., Vuong Q., Lambourne S. (2025). A Comparative Analysis of the Antioxidant Profiles Generated by the RoXstaTM System for Diverse Biological Fluids Highlights the Powerful Protective Role of Human Seminal Plasma. Antioxidants.

[37] Benedetti S., Tagliamonte M.C., Catalani S., Primiterra M., Canestrari F., Stefani S.D., Palini S., Bulletti C. (2012). Differences in blood and semen oxidative status in fertile and infertile men, and their relationship with sperm quality. Reprod. Biomed. Online.

[38] Verón G.L., Tissera A.D., Bello R., Beltramone F., Estofan G., Molina R.I., Vazquez-Levin M.H. (2018). Impact of age, clinical conditions, and lifestyle on routine semen parameters and sperm kinematics. Fertil. Steril..

[39] Kidd S.A., Eskenazi B., Wyrobek A.J. (2001). Effects of male age on semen quality and fertility: A review of the literature. Fertil. Steril..

